# Portable X-ray fluorescence (pXRF) analysis of heavy metal contamination in church graveyards with contrasting soil types

**DOI:** 10.1007/s11356-022-19676-z

**Published:** 2022-03-22

**Authors:** Charles Madden, Jamie K. Pringle, Adam J. Jeffery, Kristopher D. Wisniewski, Vivienne Heaton, Ian W. Oliver, Helen Glanville, Ian G. Stimpson, Henry C. Dick, Madeleine Eeley, Jonathan Goodwin

**Affiliations:** 1 School of Geography, Geology and Environment, Keele University, Keele, ST5 5BG Staffs UK; 2 The Keele Institute for Innovation and Teaching Excellence, Keele University, Keele, Staffordshire, ST5 5BG UK; 3 School of Physical & Chemical Sciences, Keele University, Keele, ST5 5BG Staffs UK; 4 Met Consultancy Group, Southgate House, Pontefract Road, Leeds, LS10 1SW UK; 5 School of Biosciences, University of Nottingham, Sutton Bonington Campus, Nottinghamshire, LE12 5RD UK; 6 Stoke-On-Trent Archaeology Service, Civic Centre, Stoke-on-Trent, Staffs UK

**Keywords:** Graveyards, Burials, XRF, Element analysis, Contaminated land

## Abstract

Human remains have been interred in burial grounds since historic times. Although the re-use of graveyards differs from one country, region or time period to another, over time, graveyard soil may become contaminated or enriched with heavy metal elements. This paper presents heavy metal element soil analysis from two UK church graveyard study sites with contrasting necrosols, but similar burial densities and known burial ages dating back to the sixteenth century and some possibly older than 1,000 years. Portable X-ray fluorescence element laboratory-based analyses were undertaken on surface and near-surface soil pellets. Results show elevated levels of Fe, Pb, Mn, Cr, Cu, Zn and Ca in both necrosols when compared with background values. Element concentration anomalies remained consistently higher than background samples down to 2 m, but reduced with distance away from church buildings. Element concentration anomalies are higher in the clay-rich necrosol than in sandy necrosol. Study result implications suggest that long-used necrosols are likely to be more contaminated with heavy metal elements than similar soil outside graveyards with implications for burial grounds management, adjacent populations and where burial grounds have been deconsecrated and turned to residential dwellings.

**Supplementary Information:**

The online version contains supplementary material available at 10.1007/s11356-022-19676-z.

## Introduction

Burial grounds are unique both in their natural environment, including soil type, parent material, vegetation, topography and climate, and in their anthropogenic burial numbers, styles, depths, body distributions and above-ground placement of memorials, buildings and installation of pathways and roads to access the site (Hansen et al. 2014). The mechanical disturbance via re-excavation and re-infilling of burial sites, and varying aboveground vegetation types, as well as the presence of human remains, makes graveyard soils unique and have their own necrosol soil type category (Amuno and Amuno 2014; Asare et al. 2020). Necrosols, despite increasing numbers of cemeteries and burial sites both in rural and in urban environments, are poorly understood, especially regarding potential contamination and ecological risks (Jonker and Oliver 2012). This is largely due to the complex biological and chemical processes occurring in these soils, resulting in both spatial and temporal heterogeneity of necrosols (Amuno and Amuno 2014).

Reuse of graveyard and cemetery sites for burying human remains has been happening for at least 10,000 years since Early Mesolithic times (Schulting et al. 2019). The practice of reusing existing graveyards differs spatially by country and region and relating to timing of clearing old graves before new ones are emplaced. For example, the USA generally leaves human remains untouched in situ in perpetuity, whereas in the UK it is common to have a 100-year period, by which time any direct relatives should have died before an existing graveyard can be reused (Mytum 2000), and in Germany remains can be moved when only buried for 25 years and a fresh grave is emplaced (Fiedler et al. 2009).

Soil from graveyard sites differ from the natural soil profile largely through disturbance and due to the nature of the material buried. Previous research has likened graveyards to landfills (Fiedler et al. 2012), with elevated levels of organic matter (Kim et al. 2008), embalming fluids (Chiappelli and Chiappelli 2008; Uslu et al. 2009) and creosote from coffins (Mininni et al. 2007), as well as materials from the bodies themselves including Hg and Au from teeth fillings (Fiedler et al. 2012). In a few cases, cemetery materials carried in soil water have also been found to have contaminated local groundwater supplies with pathogens, viruses and heavy metals (Konefes and McGee 2000; Matias et al. 2004; Kim et al. 2008).

Non-invasive geophysical studies in such burial grounds indicate elevated conductivity levels in grave soils (Hansen et al. 2014), with individual grave geophysical anomalies decreasing with increasing burial age, compared to background values. However, soil texture and moisture content have been shown to be major variables with sandy soils causing leaching of grave contents well beyond the grave-cut, whereas clay-rich soils tend to retain these fluids within the grave-cut itself (Pringle et al. 2012, 2016; Dick et al. 2017).

Archaeological studies have shown ancient burial ground soils to have elevated levels of heavy metal elements such as Fe, Pb, Mn and Cu (Amuno and Amuno 2014, Jonker and Oliver 2012), as well as other elements such as P and N (Bethell and Carver 1987; Asare et al. 2020), with human exposure to such toxic metals causing kidney damage (Khan et al. 2011) and links to Parkinson’s and Alzheimer’s disease (Mohod and Dhote 2013). This may have important health implications for residents in the surrounding areas as well as potential risks to the local environment via leaching into surrounding soils and groundwater (Jonker and Oliver 2012). However, there has been, to date, limited research on the soil contamination potential of cemeteries and graveyards.

X-ray fluorescence spectrometry (XRF) is an analytical technique for determining total element concentrations in a wide variety of materials and is used in environmental applications. Traditional, laboratory-based, spectrometry including inductively coupled plasma mass spectrometry, atomic emission spectroscopy and optical emission spectrometry, among others, are widely used to determine trace and heavy metal elemental concentrations in soils (Schneider et al. 2016; Messager et al. 2021). These methods have high analytical precision, but involve higher costs per sample, and lengthier sample preparation often involving heavy use of acids for digestion, data processing and analytical time.

Portable XRF (pXRF) field surveys have been shown to be effective for rapid evaluation of heavy metal soil contamination (Radu and Diamond 2009; Brent et al. 2016; Rouillon et al. 2017; Liang et al. 2018), biogeochemical mapping over mine tailings (Rincheval et al. 2019), archaeological object studies (Kasztovsky et al. 2018; Michalowski et al. 2020), marine microplastics (Turner 2017), species profiling (Nganvongpanit et al. 2016) and even lead levels in living human bones (Zhang et al. 2020). This highlights that pXRF is a powerful technique for rapid elemental analysis of soils and can be used both in situ and within the laboratory (Frahm and Doonan 2013; Goff et al. 2019). There is currently no universally agreed protocol for pXRF sample preparation related to soil analysis (Goff et al. 2019). For soil investigations, fresh soils can be challenging due to their highly variable water contents across different land uses, soil types and habitats, which can be somewhat problematic when conducting physical measurements for site comparisons, for example, electrical resistivity surveys (Jervis and Pringle 2014). This holds true for pXRF analysis, with the additional complication that the attenuation of X-rays by water is a function of the energy used to characterise the elements of interest. Low-energy X-rays are more strongly attenuated than high-energy ones; thus, for pXRF analysis, elements with lower atomic masses are more strongly impacted. Studies have shown that for every 1% increase in soil water content, there is a 1.15–1.75% decrease in reported elemental concentration for Mn through to As, whilst elements lighter than Mn are even more greatly attenuated (Parsons et al. 2013; Imanishi et al. 2010). This means that sample water content must be measured and corrected for, as light element values determined in situ will likely not be directly comparable to results of a traditional laboratory analysis (Padilla et al. 2019). Goff et al. (2019) compared different sample preparation techniques for pXRF analysis and identified that a pressed powder pellet provides best results and avoids fluorescence attenuation from field moist soil conditions.

Here we utilise the pressed pellet method to evaluate the potential heavy metal contamination of long-used (500 + years) church necrosol graveyards with different soil textures. Study objectives were therefore to (1) use pressed powder soil pellets from topsoils from 2 contrasting soils from long-established church graveyards and analyse for heavy metals using pXRF, (2) assess the respective known burial population and areal extent to quantify the respective graveyard burial histories and (3) identify potential soil and water quality implications for those living adjacent to burial grounds or living on ex-burial grounds.

## Materials and methods

### Graveyard site background

**Fig. 1:**
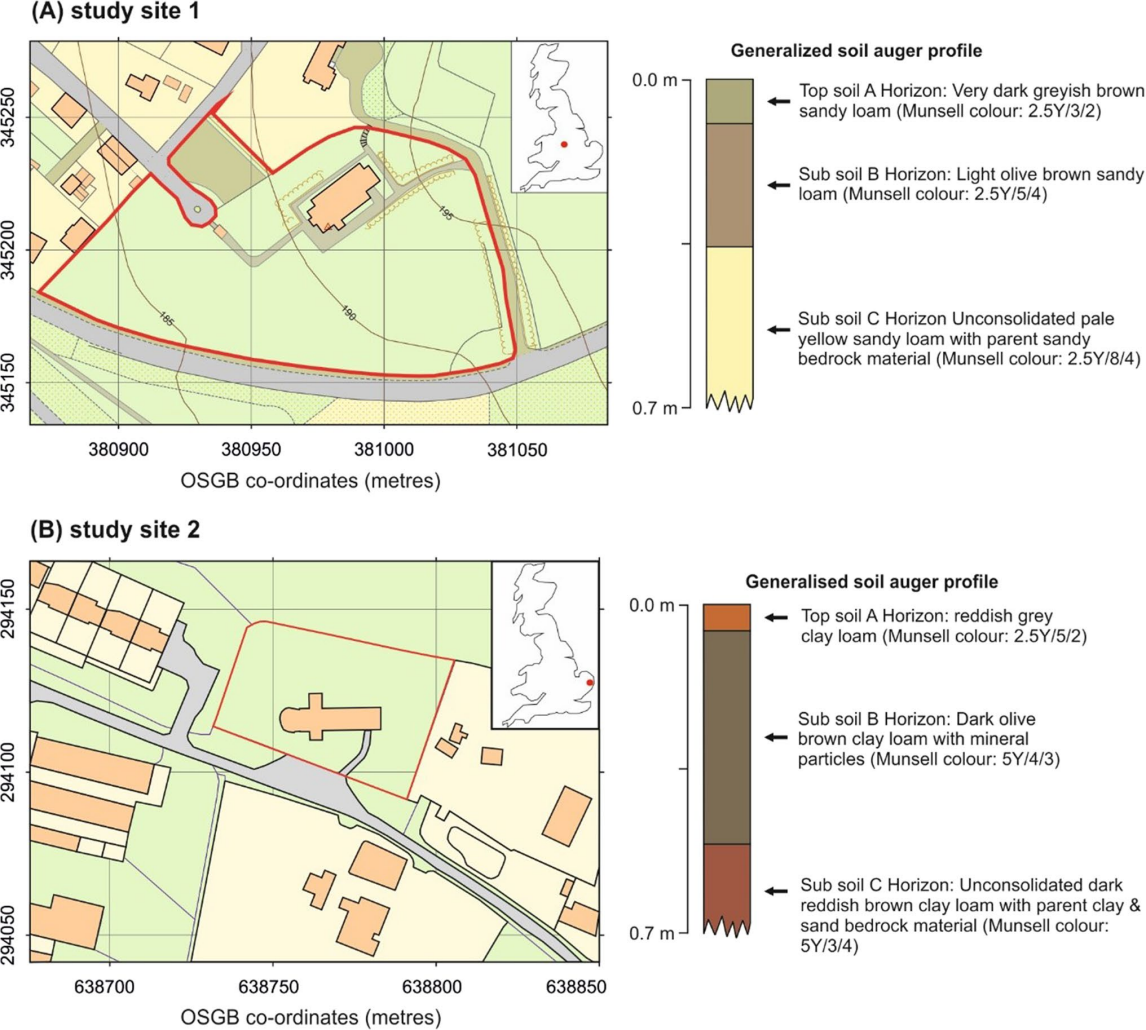
UK study site maps at **a** St. John’s Church sandy soil graveyard (red box), Keele, Staffordshire, and **b** St. Michael and All Angels’ Church clay-rich graveyard (red box), Stockton, Norfolk, with generalised soil hand-auger profile results and UK locations (inset)

Two Church of England graveyards were selected for this study, study site 1 at St. John’s Church in Keele Staffordshire, UK (Fig. 1a), and study site 2 at St. Michael and All Angels’ Church in Stockton, Norfolk, UK (Fig. 1b). These were selected as they have burial records from over 500 years ago to the present day, contrasting soil textures and bedrock types (Dick et al. 2017), different rainfall levels and geographic settings.

Study site 1 is located in the rural Keele Village in Staffordshire, situated ~ 200 m above sea level and with an average rainfall of 806 mm/year. The site was a Knights Templar church built around 1160 CE, before being taken over by the Knights Hospitaller in 1324 CE. A new church was built by the local Sneyd family in the sixteenth century, before the present sandstone church was built in 1868–1870 CE (Pevsner 1974). A desktop study, confirmed by soil auger (Fig. 1a), showed there to be a sandy loam soil overlying the Upper Carboniferous Butterton Sandstone Member of the Halesowen Formation bedrock found at ~ 2.5 m below ground level (bgl).

Study site 2 is located in the rural Stockton village in south Norfolk, situated ~ 35 m above sea level, with an average rainfall of 620 mm/year. The church of St. Michael and All Angels is present on the study site, with a Saxo-Norman-style round tower, probably dating from the thirteenth to the fourteenth century, and with later medieval flintwork additions (Knott 2005). A desktop study, confirmed by soil auger (Fig. 1b), showed there to be clay-rich soil, derived from a glacial diamicton, overlying clays and sands of the Pleistocene Beccles Formation found at ~ 2.5 m bgl.

### Known burial records

**Fig. 2:**
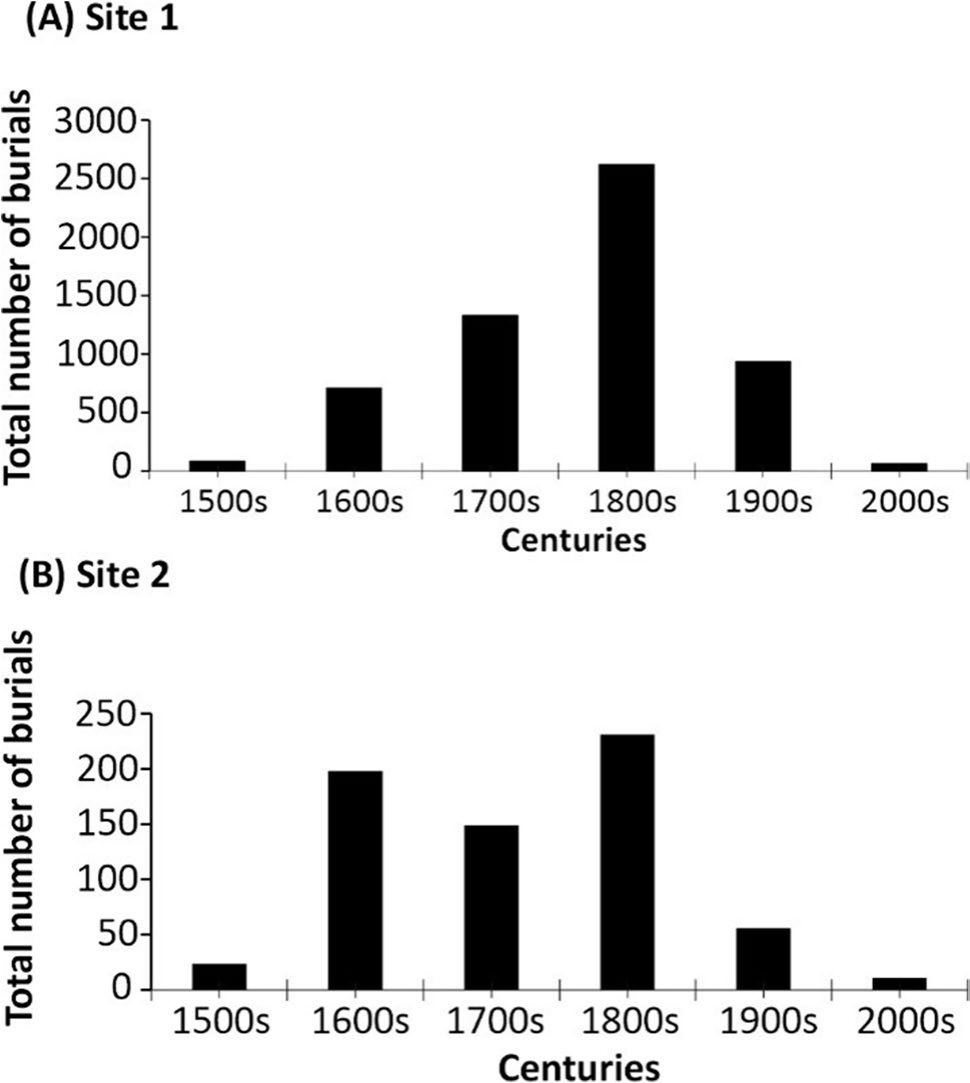
Summary plot showing known burial records of **a** 5,735 burials (1585–2018 CE) at study site 1 St. John’s Church, Keele, Staffordshire, UK, and **b** 669 burials (1561–2018 CE) at study site 2 St. Michael and All Angels’ Church, Stockton, Norfolk, UK

For site 1 at St. Johns, graveyard burial records showed there were 5,735 individuals from 1585 to 1970 CE and 1990 to 2018 CE (see Fig. 2a and Supplemental Data). The burial data gap was due a records office fire. Average yearly burials were 13/year, with a typical increase after the Industrial Revolution (the church being situated on the edge of the industrialised area of Stoke-on-Trent), before declining in the twentieth century.

For site 2 at St. Michaels, graveyard burial records there were 669 individuals from 1561–2018 CE (see Fig. 2b and Supplemental Data). Average yearly burials were 1/year, with a typical increase and decrease in burial rate with the Industrial Revolution and the twentieth century, respectively, as observed in case study 1.

When burial numbers were corrected for the areal extent of the graveyards (10,800 m^2^ for study site 1 at St. John’s and 2,330 m^2^ for study site 2 at St. Michaels), burial densities of ~ 2.2 individuals/m^2^ at study site 1 and ~ 3 individuals/m^2^ at study site 2 were determined.

Both graveyards had mostly marked earth-cut graves with headstones, with burial ages that range from the eighteenth century to the present day (Fig. 2). At study site 1, there were more multiple familial burials in the same grave which results in more variable coffin burial depths. A general review by Hart and Casper (2004) found average depths (bgl) of 1.4 m for one coffin, 1.83 m for two coffins and 2.7 m for four coffins for familial burials.

### Soil sampling and analysis

Research on optimal sampling methodologies has suggested at least five samples should be acquired in different locations on a site to gain representative results (Pye et al. 2005, 2007; McKinley and Ruffell 2007). Field reconnaissance in these two study sites determined the soil sampling areas that could be used, for example, avoiding areas of dense vegetation/trees and aboveground memorials, tarmac paths, etc., which prohibited sampling. A random number generator was then used to locate to avoid potential sampling bias within 5 m × 5 m grids at study site 1 and study site 2 respectively. Two sample arable field locations in arable fields in the same soil type were also ~ 100 m away from each graveyard (Fig. 2) which were used to act as a control to quantify local soil background element values.

### pXRF surface soil sampling

Overlying vegetation was firstly removed to expose the soil at 33 and 31 sampling locations at study sites 1 and 2, respectively, which was then checked for pebbles or vegetation which would contaminate the measurement (Fig S1).

Approximately 250 g of surface soil from the top 5 cm was collected from each sampling location at both study sites, bagged, labelled and stored at 4 °C. A subset of samples was also taken for routine soil characterisation analysis, initially including determination of average electrical conductivity, soil pH and water contents following standard soil characterisation methodologies.

To create the pressed powder pellet, surface soil samples were oven-dried for 24 h at 105 °C before being hand-ground using a pestle and mortar to pass through a 63-μm sieve. The sample was then mixed with 1.5 mL of polyvinylpyrrolidone-methylcellulose binding agent and mechanically pressed under 10-tn pressure (Fig S1) into a homogenous flat-cylinder pellet, before being oven-dried for a further 24 h at 105 °C to remove any soil water influence on signal strength (Kalnicky and Singhvi 2001). Each pellet (Fig S1) was then analysed using the NITON XL3t 900 pXRF Analyzer in its laboratory holder, using a 5-min measurement time, chosen as a compromise between analytical time and instrumental precision (see Pringle et al. 2022). The instrument employs four sub-filters (Main, Low, High, Light), each targeting a specific range of elements. Each of these filters was allotted an equal proportion of the overall analytical time. Analytical precision, expressed as two standard deviations of repeat analyses of the NIST2709 international reference material, was 1% for Fe and Ti, 2% for Ca and < 5% for Mn, Zn and Cr. Precision for Pb was 14%, and 28% for Cu and As, due to low concentrations. All analytes with the exception of As and Cr were quantified using a user calibration, calibrating the instrument using a range of international reference materials (AGV-1, RGM-1, QLO-1, NIM-S and DR-N). For these analytes, accuracy (expressed as percentage deviation from the recognised values of the NIST2709 international reference material) was quantified at 1% for Fe and Ti; < 6% for Mn, Zn and Ca; and < 15% for Cu. By contrast, analysis of As and Cr was found to yield more accurate results using the in-built, factory-standard Niton calibration of the instrument, and yielded accuracies of 32% and 18%, respectively.

### Soil depth profile

To determine element variation at depth, 0.75 m (*n* = 3) soil cores were taken using a handheld slimline hand auger at 32 and 31 sampling locations at study site 1 and study site 2 graveyards respectively. Cores were split into 0.25-m intervals (0–0.25 cm, 0.26–0.50 cm and 0.51–0.75 cm respectively) (Fig S1). The resulting soil samples were then labelled and stored in polyethylene bags at 4 °C until soil pellets from each of the listed soil depth intervals were generated using the procedure as already detailed above and then each pXRF analysed in the laboratory for a 5-min measurement time as previously described.

### Single site deep soil core

A grave digger was hand-digging a new grave in study site 1 whilst soil sampling was being conducted, so opportunistically the research team were able to collect deeper subsoil samples. Here, ~ 250 g of soil samples was collected every 0.25-m down to 2-m depth on the south end of the empty grave (Fig S1). The resulting soil samples were labelled and stored in polyethylene bags at 4 °C until soil pellets were generated and pXRF analysed in the laboratory for a 5-min measurement time procedure as already detailed.

## Results

### Basic soil characterisation

The samples had routine soil characterisation analysis, including determination of electrical conductivity, pH and soil moisture content following standard methodologies. Study site 1 electrical conductivity (EC) of soils recorded an average of 47 μS/cm (28 SD), with study site 2 recording an average EC of 99 μS/cm (49 SD). Study site 1 pH of soils displayed an average of 6.3 (0.8 SD), with study site 2 recording an average of 7.8 (0.8 SD). Study site 1 soil moisture content ranged from 8 to 27% (average 14.1%), with study site 2 soil moisture content ranging from 13 to 27% (average 21%).

### pXRF surface soil sampling

Our results show clear differences in element concentrations in the soil samples measured, when compared to the background control samples, within each study site graveyard and between the two graveyards summarised in Tables 1 and 2.

**Table 1:** Descriptive statistics of heavy metal element pXRF concentration laboratory soil pellets over 5-min measurement duration, acquired from study site 1. Raw data in Supplementary Material. *Av* average, *n* number of analyses

Element	Laboratory (*n* = 32) dry soil pellets (mg/kg)				Control soil (*n* = 2) dry soil sample pellets (mg/kg)
	Min	Av	Max	SD	Av
Fe	17,011	35,284	59,954	9107	40,972
Pb	69	188	742	158	51
As	4	8	18	3	8
Mn	77	1,177	2409	483	1104
Zn	49	115	319	61	70
Cr	31	58	127	19	99
Cu	23	39	99	15	–
Ti	1504	3457	4609	596	5333

**Table 2:** Descriptive statistics of heavy metal element pXRF concentration laboratory soil pellets over 5-min measurement duration, acquired from study site 2. Raw data in Supplementary Material. *Av* average, *n* number of analyses

Element	Laboratory (*n* = 31) dry soil pellets (mg/kg)				Control soil (*n* = 1) dry soil sample pellets (mg/kg)
	Min	Av	Max	SD	Av
Fe	6938	26,385	55,546	8435	17,705
Pb	39	284	2032	414	28
As	3	8	21	3	8
Mn	179	381	1129	208	486
Zn	73	493	5865	1098	95
Cr	8	55	80	18	36
Cu	22	38	122	23	13
Ti	928	3455	4563	1043	3505

**Fig. 3:**
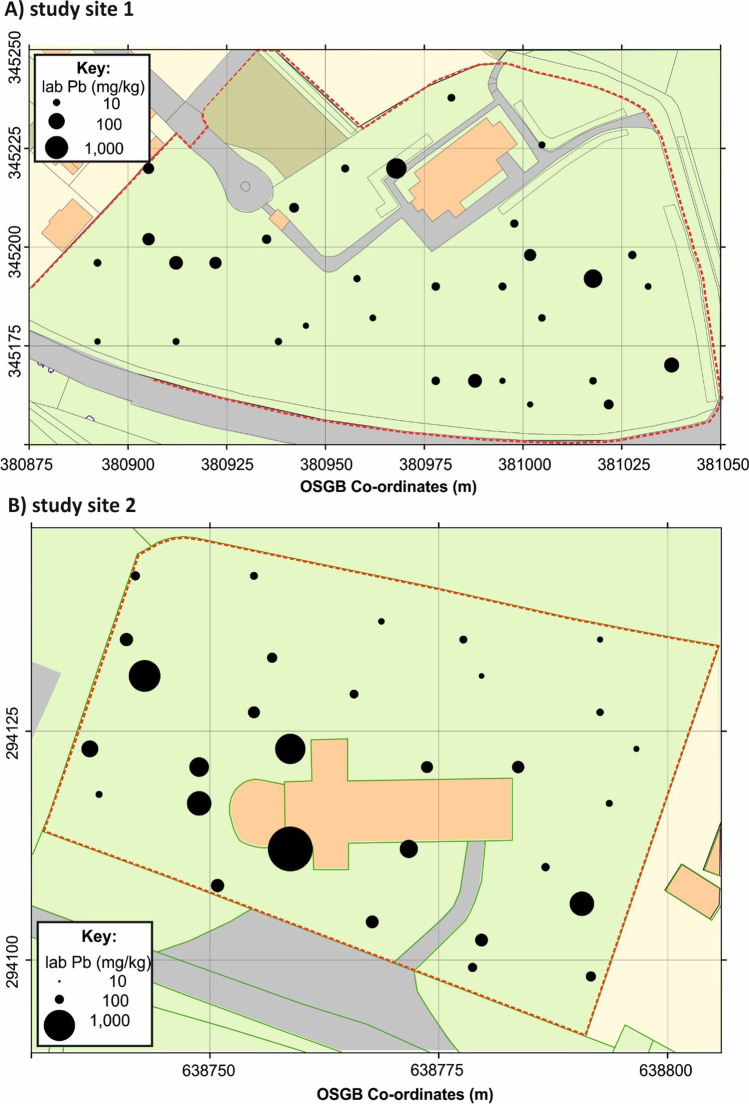
Laboratory soil pellet lead element pXRF concentrations for **a** study site 1 and **b** study site 2 respectively. Dashed line marks graveyard extents

At study site 1 with the sand-rich soil, surface soil pXRF measurements showed Pb element concentrations varying from 69 mg/kg up to a maximum of 742 mg/kg with an average of 188 mg/kg (Table 1), with relatively higher concentrations adjacent to the church itself (Fig. 3a). Control soil Pb element concentration average was only 30 mg/kg. Mn also had high concentrations, varying from 77 mg/kg up to a maximum of 2,409 mg/kg with an average of 1177 mg/kg (Table 1), with background Mn soil concentration average of 798 mg/kg. As had relatively low concentrations, varying from 4 up to 18 mg/kg with an average of 8 mg/kg, the same concentration as the control soil samples (Table 1). Cu had an average of 29 mg/kg with control soil sample being below detection levels (Table 1). Ca had an average of 19,317 mg/kg with control soil sample averages being 1738 mg/kg.

At study site 2 with the clay-rich soil, surface soil pXRF measurements showed very high Pb concentrations, varying from 38 mg/kg up to a maximum of 2,310 mg/kg with an average of 317 mg/kg (Table 1), with higher concentrations adjacent to the church (Fig. 3b). Control soil Pb element concentration averages was only 28 mg/kg. Zinc had also high concentrations, varying from 64 up to 6,528 mg/kg, with an average of 532 mg/kg (Table 2). The Zn distribution across the graveyard was more varied when compared to the lead distributions, with background Zn soil average of 67 mg/kg. Arsenic had similar concentrations as background control samples, varying from 3 up to 21 mg/kg with an average of 8 mg/kg. Cu had an average of 26 mg/kg with control soil sample being 13 mg/kg (Table 1). Ca had an average of 37,709 mg/kg with control soil sample averages being 4492 mg/kg.

Comparing element concentrations between graveyards, Pb concentrations were higher in the clay soil of study site 2 compared to the sandy soil of study site 1 which has been reported elsewhere (see Yuan et al. 2014).

**Fig. 4:**
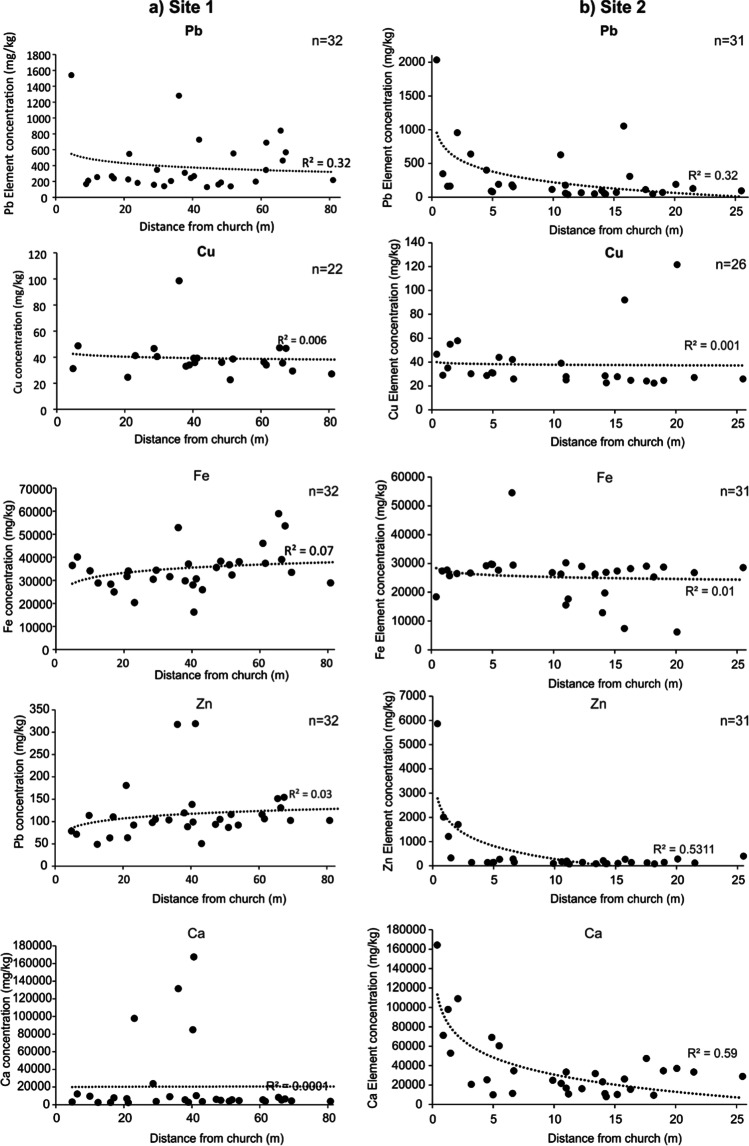
Graphs showing selected element soil pellet pXRF concentrations with distance from **a** study site 1 and **b** study site 2. Note logarithmic trendline with *R*^2^ fit is shown

As soil sample locations were geospatially referenced, elements could be compared directly with the analysis of distance from the respective churches at each study site (Fig. 4). Pb, Cu and Zn elements showed decreasing concentration trends with increasing distance from the church buildings but these were not statistically significant. Ca levels were recorded at very high concentrations adjacent to site 2 church building (> 100,000 mg/kg) when compared to ~ 5,000 mg/kg average control soil values. This was expected due to the lime plaster building construction that was present here but still useful to evidence.

### Variations down a soil depth profile

**Fig. 5:**
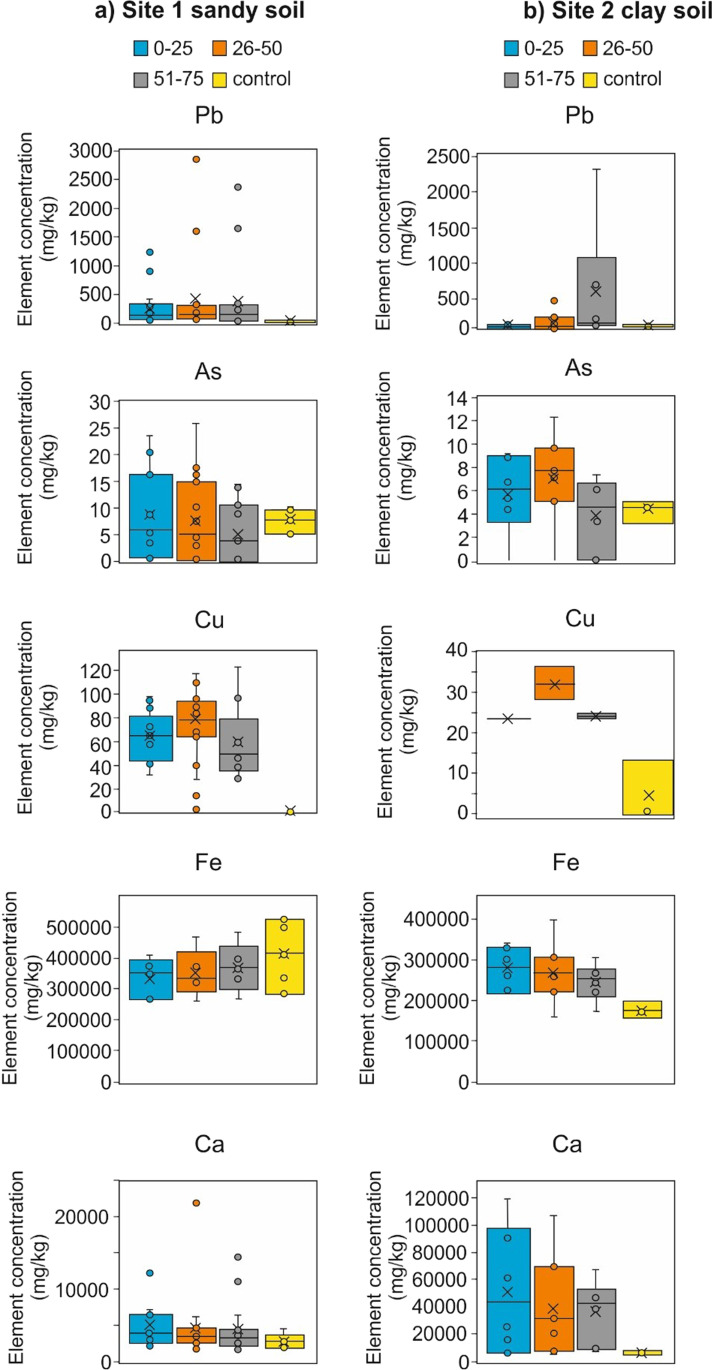
Box-whisker graph plots of selected element pXRF concentrations from soil auger pellets from the different depths investigated (see keys) from **a** study site 1 sandy soil, and **b** study site 2 clay soil graveyards

At the sand-rich soil study site 1, Pb concentrations were consistently high, averaging 287 mg/kg at 0–0.25 m, 434 mg/kg at 0.26–0.5 m and 381 mg/kg at 0.51–0.75 m bgl (Fig. 5), which contrasts with 30-mg/kg control levels. As concentrations were low averaging ~ 10 mg/kg that was similar to background values (Table 3). Cu concentrations were ~ 64 mg/kg through the soil depth profile with control soil values being below detectable levels. Fe concentrations increased with soil depth in both the graveyard and control values and Ca decreased with soil depth.

**Table 3:** Descriptive heavy metal element pXRF concentration statistics of laboratory soil depth range pellets over 5-min measurement duration, acquired from study site 1 sandy soil. *Av* average, *n* number of analyses

Selected element	*0–0.25 m bgl* (*n* = 15) dry soil pellets (mg/kg)					*0.26–0.5 m bgl* (*n* = 15) dry soil pellets (mg/kg)					*0.51–0.75 m bgl* (*n* = 15) dry soil pellets (mg/kg)				
	Graveyard (*n* = 15)				Control (*n* = 2)	Graveyard (*n* = 15)				Control (*n* = 2)	Graveyard (*n* = 15)				Control (*n* = 2)
	Min	Av	Max	SD	Av	Min	Av	Max	SD	Av	Min	Av	Max	SD	Av
26,500	33,345	41,229	6560	39,452	26,042	35,116	46,911	76,911	43,023	26,715	36,874	48,176	7915	40,441	26,500
9	287	1236	342	41	30	434	2854	770	32	9	381	2375	686	18	9
4	12	24	7	9	3	11	26	7	8	4	9	14	4	6	4
551	1399	2272	489	687	327	1698	2725	764	899	256	1473	2247	638	806	551
51	106	187	35	80	53	90	178	34	72	31	71	120	21	60	51
53	105	170	31	79	50	130	199	44	84	43	109	185	46	82	53
31	64	97	21	-	28	77	117	26	-	29	60	123	32	-	31
3139	3571	4346	475	4864	3110	3758	4484	539	5671	3334	3746	4697	556	5466	3139

At the clay-rich soil study site 2, Zn and Pb element concentrations increase with increasing soil depth bgl, with Pb in particular increasing, averaging from 39 mg/kg at 0–0.25 m, 142 mg/kg at 0.26–0.5 m to 608 mg/kg at 0.51–0.75 m bgl (Fig. 5), which contrasts with the 31-mg/kg control sample concentrations. As concentrations were low and similar to the control average of 4–7 mg/kg (Table 4). Cu concentrations were ~ 12–21 mg/kg through the soil depth profile with control soil values being below detectable levels. Fe and Ca concentrations decreased with soil depth and Fe values were about twice as high as control soil Fe values.

**Table 4:** Descriptive heavy metal element pXRF concentration statistics of laboratory soil depth range pellets over 5-min measurement duration, acquired from study site 2 clay-rich soil. *Av* average, *n* number of analyses

Selected element	*0–0.25 m bgl* (*n* = 6) dry soil pellets (mg/kg)					*0.26–0.5 m bgl* (*n* = 6) dry soil pellets (mg/kg)					*0.51–0.75 m bgl* (*n* = 6) dry soil pellets (mg/kg)				
	Graveyard (*n* = 7)				Control (*n* = 1)	Graveyard (*n* = 7)				Control (*n* = 1)	Graveyard (*n* = 7)				Control (*n* = 1)
	Min	Av	Max	SD	–	Min	Av	Max	SD	–	Min	Av	Max	SD	–
Fe	22,407	29,087	34,088	4807	19,908	22,168	28,635	39,769	6090	15,848	21,965	26,046	30,232	3102	17,360
Pb	20	43	92	28	19	28	134	417	149	26	46	637	2313	967	47
As	6	8	9	2	4	7	9	12	2	5	6	7	7	1	3
Mn	276	345	384	51	425	140	283	412	107	389	208	261	311	40	427
Zn	65	110	256	82	59	76	126	292	84	26	46	637	2313	967	47
Cr	44	95	234	79	46	43	66	87	15	29	47	51	59	5	33
Cu	23	23	24	0.2	-	28	32	36	6	-	24	24	24	n/a	25
Ti	2842	4103	4983	882	3798	2863	4013	5522	867	3472	2370	3136	3653	526	3618

### Single-site deep soil core

**Fig. 6:**
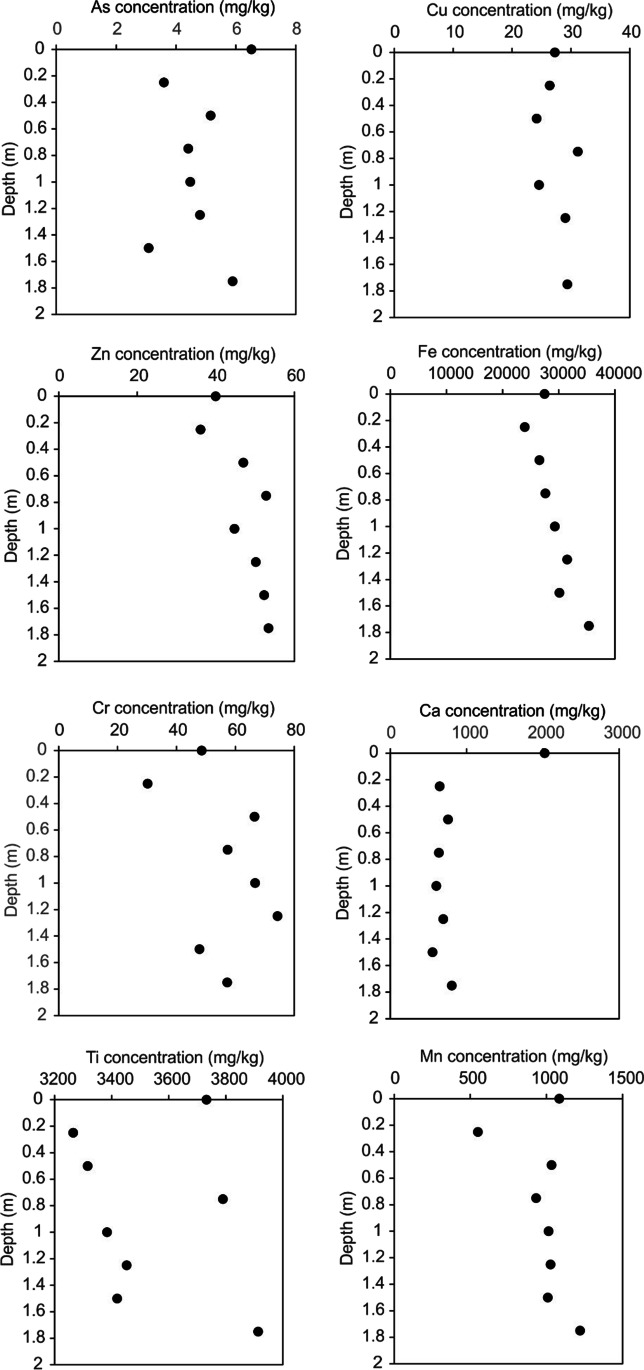
Graphs of selected element pXRF concentrations against the empty grave depths bgl at study site 1 St. Johns graveyard sandy soil graveyard, Keele, Staffordshire, UK

Here at the sandy soil study site 1, Fe, Zn, Mn and Cr element concentrations generally increased with soil depth bgl with Cu constant and As and Ca decreasing with soil depth (Fig. 6). Ca concentrations reduced with depth to 600 mg/kg at 2 m bgl, slightly higher than the national average of 400 mg/kg for sandy rural soils as given by Ross et al. (2007). These results (Table 5) reinforce the soil auger profile results although note this was only one empty grave sample at one location within the study site 1 graveyard.

**Table 5:** Descriptive heavy metal element pXRF concentration statistics of laboratory soil depth range pellets over 5-min measurement duration, acquired from study site 1 St. John’s graveyard, Keele, Staffordshire, Norfolk, UK

Depth (m)		Selected heavy metal element concentrations (mg/kg)							Ca (mg/kg)
	Fe	Pb	As	Mn	Zn	Cr	Cu	Ti	
0.0	40,125	91	11	1599	97	61	46	3932	3565
0.25	50,425	29	11	1085	88	90	33	6066	2856
0.5	25,350	28	bdl	1106	39	59	–	4298	3162
0.75	25,494	17	4	822	37	40	–	3890	2720
1	33,656	50	4	804	51	71	28	4037	2917
1.25	33,646	12	5	689	42	70	–	3820	2356
1.5	24,730	11	4	1042	33	53	–	3495	1815
1.75	33,779	11	4	1201	41	59	26	3753	1830
2.0	41,692	13	5	1376	44	64	38	3926	1838

## Discussion

Here, we aimed to evaluate the potential heavy metal contamination of long-used (500 + years) church necrosol graveyards with different soil types. The graveyard datasets acquired from surface soil (0–0.05 m), shallow soil depths (> 0.75 m) and single deep soil (> 2 m) (at study site 1 only) all showed heavy metal concentrations were not only higher than background control soil samples taken ~ 100 m away from both graveyards (Tables 1–5), but were also higher than average concentrations observed in the soils of England and Wales (Ross et al. 2007). Measured Pb and As element concentrations were well above the threshold level (75 mg/kg) identified as potentially resulting in ecotoxicological effects (deVries et al. 2007). In many of the graveyard soil sampling points examined, Pb concentrations were also above the predicted ‘no effect’ concentrations of 166 mg/kg and 212 mg/kg reported by Smolders et al. (2009) and the European Chemicals Agency (ECA-Ecotoxicological Summary for lead), respectively. Our paper shows heavy metals were present in both study site soils at much higher concentrations than those found in other studies, with Neckel et al. (2016), for example, only recording Pb values up to 127 mg/kg (Table 6). Fiedler et al. (2012) graveyard study evidenced comparable Pb levels from this study to an uncovered coffin (Table 6). It should be noted that As concentrations were generally low at both study sites and did not exceed either control values or WHO soil standards of ~ 10 mg/kg, even though it is not very mobile in alkaline soil (Fiedler et al. 2012).

**Table 6:** Summary statistics of this study soil pellet pXRF results compared to other studies and *mean UK soil values from Ross et al. (2017)

Element	Case study 1 sandy soil av. pellets (mg/kg)	Case study 1 control sandy soil (mg/kg)	Case study 2 clay soil av. pellets (mg/kg)	Case study 2 control clay soil (mg/kg)	Neckel et al. (2016) 3 cemeteries av. surface grave soil (mg/kg)	Fiedler et al. (2012) coffin material (mg/kg)	*Mean UK rural soil (mg/kg)
Pb	188	30	284	31	34	492	52.5
As	8	8	8	4	–	4.6	10.9
Mn	1178	798	381	413	304	–	612
Zn	115	71	493	76	105	821	81.2
Cr	58	81	55	36	25	26.2	34.4

There were also large variations of measured heavy metal element concentrations within each graveyard, with the highest concentrations being found generally in soil samples taken adjacent to church buildings. This would suggest building materials would be a major source of heavy metals found in the adjacent soils such as Pb flashings, particularly as element concentrations decreased with increasing spatial distance from the churches. Other elements also showed this trend such as Ca, which may suggest a higher burial density nearest the church and corresponding release of elements from coffins as Fiedler et al. (2012) measured from graveyard coffins in Germany (Table 6). Generally, soil samples showed the same trend of decreasing element concentrations with increasing soil depth, except for Pb at study site 1 with sandy soil which had consistently high lead values down to 0.75 m. As given in the introduction, the coffins themselves are often a source of metal contamination so burial concentration should be a factor when looking at characterising necrosols.

Comparing the two graveyard study sites, although the number of known burials and graveyard areal extent was different, the actual burial density was similar (~ 2.5 m^2^), so element concentration differences may be due to the different soil type, with clay-rich soils having greater reactive surface areas which can bind metals and other mineral and organic substances in the environment (Weil and Brady 2017). Surface soil (and down to 0.25 m bgl) heavy metal concentrations were generally higher in the clay soil at study site 2, when compared to the sandy soil at study site 1, suggesting elements are less mobile in the low porosity/permeability clay soils. In contrast, higher element concentration values occur in deeper soils in the sandy study site 1 but unfortunately deeper samples than 0.75 m bgl were not collected from the clay soil at study site 2 so it cannot be stated definitively that higher element levels are not present at depth in this graveyard.

This study has important implications for managing both historical and contemporary burial grounds, in relation to re-use and potential environmental and ecological contamination impacts from burial sites. Depending upon the soil type, as evidenced here, mobile heavy metals may leach away from the graveyard area itself and potentially to nearby surface and groundwater supplies if the geological conditions are suitable for this, as detailed by Oliveira et al. (2012) and Matias et al. (2004) evidenced from a Portuguese graveyard study and nearby water borehole results. Finally, a number of closed churches, graveyards and cemeteries are being deconsecrated and turned into residential dwellings with bodies being commonly left rather than being exhumed and reburied in nearby burial grounds. Soil analysis for heavy metal concentrations in such grounds would be highly recommended especially if people living in these areas wanted to grow edible produce which could bioaccumulate these heavy metals in their tissues. Further research is needed on these converted site to assess this important environmental and human health contamination risk.

### Future work

Whilst one empty grave at the study site 1 was able to be sampled, it would obviously be advantageous to sample grave soil deeper and ideally adjacent to coffins themselves as per Fiedler et al. (2012) adipocere study. Other burial ground types, for example green or ‘natural’ burials, are becoming increasingly popular globally, with 270 UK sites being built between 1993 and 2015 alone (Yarwood et al. 2015). These generally have lower burial densities, when compared to cemetery/graveyard burial grounds, biodegradable receptacles (e.g. shrouds, cardboard or wicker-based), but involve more shallow or even vertical burials (Kim et al. 2008). The study of element mobility in graveyard soils should also be undertaken to determine which necrosols are likely to be more contaminated. These factors would suggest early decomposition stages releasing more fluids, including embalming fluids, into the surrounding soil, when compared to more traditional burials, with accompanying increased surrounding soil contamination, but little research has been undertaken on this to-date.

## Conclusions

This paper provides two case studies of long-used (500 + years) burial grounds, UK church graveyards in this case, whose necrosols are contaminated by heavy metals. Here we used pXRF to determine the spatial extent (distance and depth) of heavy metal contaminations, and highlight its use for rapid data collection across different environmental samples. In particular, we identified Pb concentrations were well in excess of current environmental guidelines, although these concentrations are not uniformly distributed, both in extent across the graveyards and in depth below ground level. The highest levels of contamination are in the top 0.25 m and adjacent to church structures, potentially due to high burial concentrations and/or due to relict church materials being incorporated into the soils. This will be important for burial ground management, those living adjacent to burial grounds, potential surface/groundwater contamination and where burial grounds have been deconsecrated and turned into residential dwellings.

This paper is limited by only studying two UK graveyards, albeit long-used with different soil types, and by the numbers of soil samples collected, analysed and measured. However, the implications for other church graveyards to be similarly contaminated is clear. More accurate analytical equipment should be used to refine these initial results and obtain absolute element measurements to further validate the use of pXRF in determining heavy metal contamination as this could help speed up and reduce costs for soil testing in potentially contaminated sites. Here, we highlight graveyards are potential repositories for heavy metals but could also be possible stores for other emerging environmental contaminations too, such as pharmaceuticals which may persist in graveyard soils after decomposition. Further research is needed to explore other graveyards, cemeteries, green burials and other burial grounds with different burial ages, in other soil types, as well as collecting soil within and adjacent to graves in order to assess environmental contamination risks as well as future environmental sustainability.

## Supplementary Information

Below is the link to the electronic supplementary material.

Supplementary file1 (DOCX 556 KB)

## Data Availability

pXRF data and burial records for the two respective case study sites are available on Keele’s eRepository, the DOI link of which is: http://doi.org/10.21252/sjkk-w810
