# Supplementary material for: Portable X-ray fluorescence (pXRF) analysis of heavy metal contamination in church graveyards with contrasting soil types
Source: Environ Sci Pollut Res Int. 2022 Mar 22;29(36):55278–92. doi: 10.1007/s11356-022-19676-z (PMC9356940; doi:10.1007/s11356-022-19676-z)
Supplement: Supplementary file 1 — Supplementary file1 (DOCX 556 KB) [file 11356_2022_19676_MOESM1_ESM.docx]

***
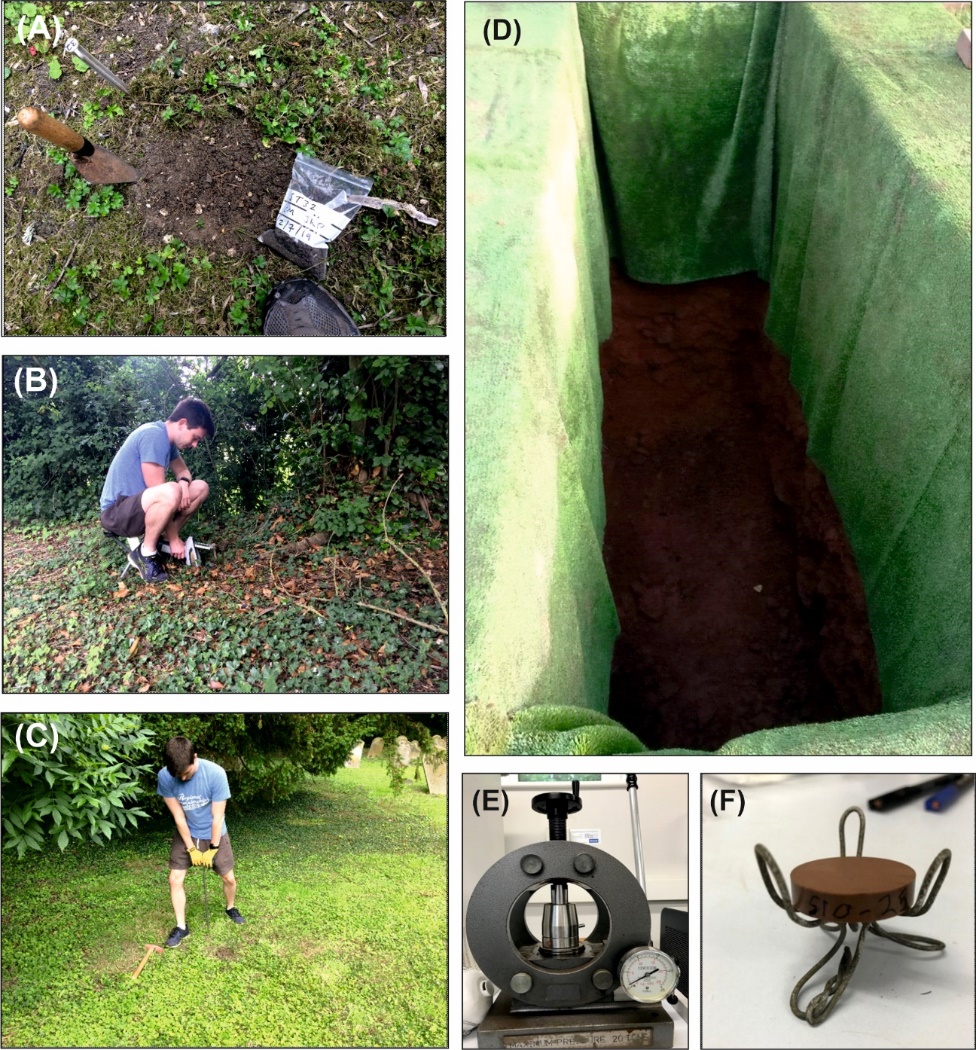
***

**Figure S1.** (A) selected sample location with overlying vegetation/pebbles cleared and sample bag shown. (B) Field pXRF 120 s analysis of a sampled surface graveyard soil location. (C) 0.75 m soil auger used 3x at each location to collect 0-25cm, 26-50cm and 51-75cm depth ranges soil samples. (D) pre-dug grave that was able to be sampled every 0.25 m down to 2 m bgl. (E) 20 Tn soil press used to produce (F) soil pellet, see text for details.
